# Possible Transmission Mechanisms of Mixed *Mycobacterium tuberculosis* Infection in High HIV Prevalence Country, Botswana

**DOI:** 10.3201/eid2605.191638

**Published:** 2020-05

**Authors:** Yeonsoo Baik, Chawangwa Modongo, Patrick K. Moonan, Eleanor S. Click, James L. Tobias, Rosanna Boyd, Alyssa Finlay, John E. Oeltmann, Sanghyuk S. Shin, Nicola M. Zetola

**Affiliations:** University of California, Los Angeles, Los Angeles, California, USA (Y. Baik);; Botswana–Upenn Partnership, Gaborone, Botswana (C. Modongo, N.M. Zetola);; US Centers for Disease Control and Prevention, Atlanta, Georgia, USA (P.K. Moonan, E.S. Click, J.L. Tobias, R. Boyd, A. Finlay, J.E. Oeltmann);; US Centers for Disease Control and Prevention, Gaborone (R. Boyd, A. Finlay);; University of California, Irvine, Irvine, California, USA (S.S Shin)

**Keywords:** Tuberculosis and other mycobacteria, bacteria, Mycobacterium tuberculosis, Botswana, HIV, tuberculosis, genotyping, mixed infection

## Abstract

Tuberculosis caused by concurrent infection with multiple *Mycobacterium* *tuberculosis* strains (i.e., mixed infection) challenges clinical and epidemiologic paradigms. We explored possible transmission mechanisms of mixed infection in a population-based, molecular epidemiology study in Botswana during 2012–2016. We defined mixed infection as multiple repeats of alleles at >2 loci within a discrete mycobacterial interspersed repetitive unit–variable-number tandem-repeat (MIRU-VNTR) result. We compared mixed infection MIRU-VNTR results with all study MIRU-VNTR results by considering all permutations at each multiple allele locus; matched MIRU-VNTR results were considered evidence of recently acquired strains and nonmatched to any other results were considered evidence of remotely acquired strains. Among 2,051 patients, 34 (1.7%) had mixed infection, of which 23 (68%) had recently and remotely acquired strains. This finding might support the mixed infection mechanism of recent transmission and simultaneous remote reactivation. Further exploration is needed to determine proportions of transmission mechanisms in settings where mixed infections are prevalent.

Tuberculosis (TB) caused by concurrent infection with multiple strains of *Mycobacterium tuberculosis* during 1 episode is commonly referred to as mixed infection. In 1972, Canetti et al. suggested the concept of mixed infection of exogenous reinfection of nonprimary TB among elderly patients in France (*1*). Their observation was followed by phage typing of cultured isolates from patients with concurrent disease in multiple organ sites observed during clinical practice in North America, mixed cultures among Eskimo patients during the mid-1970s (*2*,*3*), and cultures collected during outbreak investigations in the 1980s and 1990s (*4*,*5*). However, more recent applications of advanced molecular tools suggest mixed infection might occur more frequently than initially expected (*6*,*7*). This possibility led to many research studies of mixed infection, which found that mixed infection is associated with poor treatment outcomes (*6*,*8*), including acquisition of multidrug-resistant TB (*7*,*8*). Mixed infection research contributed to the discovery that exogenous reinfection was responsible for a substantial portion of incident TB, implying incomplete protection from a primary infection in subsequent infections (*9*,*10*).

Despite the clinical importance of mixed infection, its potential leading mechanisms of transmission have not been examined using empirical data. Infections caused by multiple *M. tuberculosis* strains can occur after simultaneous transmission of multiple strains during a single transmission episode (i.e., the index patient transmits multiple strains) or by sequential infections of >2 strains acquired at different times, resulting in superinfection (*10*). So far, transmission mechanisms of mixed infection and its population-level effect have been explored only hypothetically (*9*,*11*). Research on the transmission mechanisms for mixed infection with empirical data might improve understanding of *M. tuberculosis* dynamics and designing effective TB control interventions (*12*). Our objective was to explore possible transmission mechanisms leading to mixed *M. tuberculosis* infections by comparing genotypes and spatial proximity of all detected *M. tuberculosis* strains.

## Methods

### Study Setting

This analysis was part of a population-based, molecular epidemiology study in Botswana (the Kopanyo Study). The study design and methods were previously described (*13*). In brief, the study recruited and enrolled patients with newly diagnosed TB at 30 TB and HIV clinics during 2012–2016. Behavioral, clinical, and demographic information (including residential address at enrolment) were collected during medical record abstraction and standardized patient interview. Sputum collected from participants underwent smear-microscopy, culture, drug-susceptibility testing, and 24-locus mycobacterial interspersed repetitive unit–variable-number tandem-repeat (MIRU-VNTR) genotyping using a standard international protocol (*14*), when applicable.

### Definition of Mixed Infection

**Figure 1:**
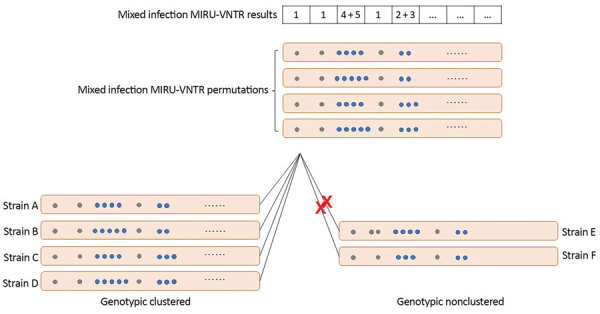
Mixed-strain infection MIRU-VNTR permutations and genotype cluster/noncluster examples of *Mycobacterium tuberculosis* (the Kopanyo Study), Botswana, 2012–2016. On the basis of mixed-strain MIRU-VNTR patterns, all possible permutations at each of multiple allele loci were considered. The MIRU-VNTR result of each strain in a possible permutation set was compared with that of all strains identified in the study. Assuming numbers of tandem repeats at other 19 loci are identical, 4 genomes (strains A–D) in the genotype cluster example (bottom left) have matched tandem repeats at the presented 5 loci of tandem repeats in the mixed-strain infection. Strains E and F in the genotype noncluster example have nonmatched tandem repeats at the second and third locus, respectively. MIRU-VNTR, mycobacterial interspersed repetitive unit–variable-number tandem-repeat.

MIRU-VNTR genotyping counts the numbers of tandem repeats at the selected loci, which are unique in different strains of *M. tuberculosis*. We defined mixed infection as multiple allele repeat numbers (e.g., double allele) at >2 loci within a discrete MIRU-VNTR result (*10*). We defined possible mixed infection as multiple allele repeat numbers at 1 locus within a discrete MIRU-VNTR result and single infection as a discrete MIRU-VNTR result with single alleles at all 24 loci (Figure 1; Appendix Tables 1, 2).

### Definition of Genotype Cluster

We defined TB genotype clusters as >2 patient isolates with exact match 24-loci results, suggesting recently acquired strains (*12*,*15*). We considered genotype results that matched no other patient isolate results in the dataset nonclustered, suggesting remotely acquired strains (*12*,*15*). To identify putative mixed infection *M. tuberculosis* genotype clusters, we compared MIRU-VNTR results for each mixed infection patient to MIRU-VNTR results of all other *M. tuberculosis* strains, considering permutations of each repeat number at multiple allele loci. We also considered 24-loci results to be nonclustered if no permutation of the mixed MIRU-VNTR result matched any other study strain; if >1 permutation of the mixed MIRU-VNTR result matched any other study strain, we considered it to be clustered. When we considered all permutations at each double allele locus, if >1 permutation accounting for each repeat number at each locus matched another study strain but no permutation accounting for the alternate repeat number at each locus matched another study strain, we considered it to be evidence of simultaneously clustered and nonclustered strains. For example, if the patient isolate results had repeat numbers 4 and 5 at the third locus, the matched *M. tuberculosis* strain’s MIRU-VNTR results should include repeat numbers 4, 5, or both at the same locus (Figure 1). We excluded patients with isolates that had missing or incomplete MIRU-VNTR results. We reviewed all laboratory procedures (i.e., sputum collection and processing, culture isolation and storage, DNA abstraction and storage, and MIRU-VNTR batching processes) to identify potential points of cross-contamination or mishandling. We reviewed all laboratory registries and electronic databases to record processing and reporting dates for all patient isolates.

### Classification of Mixed Infection Mechanisms

On the basis of the genotype cluster analysis, we classified patients with mixed infection into 1 of 3 categories: 1) simultaneous reactivation of >2 remotely acquired strains if no mixed infection MIRU-VNTR permutations accounting for multiple different repeat numbers at each locus matched any other study strain; 2) infection from a recently acquired strain and simultaneous reactivation of a remotely acquired strain if >1 permutation accounting for 1 repeat number at each locus matched another study strain but no permutation accounting for the other repeat number at each locus matched any other study strain; and 3) rapid progression of >2 recently acquired strains if >1 permutation accounting for each repeat number at each double allele locus matched another study strain.

### Statistical Analyses

For each mixed infection MIRU-VNTR result, we wrote a loop function using SAS (SAS Institute Inc., https://www.sas.com) to compare tandem numbers from the first locus to 24th locus with all other MIRU-VNTR results locus by locus. When discrepancies existed between tandem numbers at a locus, the locus was flagged. We counted the number of flags after 24 loci were compared. If all numbers matched, the number of flags was 0; if no loci matched, the number was 24. We used the number of flags to classify the degree to which the MIRU-VNTR pattern matched that of the mixed infection MIRU-VNTR result. At the end of the loop function, we created a subset dataset with all MIRU-VNTR results by descending order of the number of exactly matched loci (from 0 for exactly matched at all 24 loci), for each mixed infection MIRU-VNTR result.

We calculated simple frequencies and proportions for the main outcomes (mixed infection, possible mixed infection, and single infection) stratified by patient sex, HIV status, and residential address. Primary residential address of each patient was geocoded and mapped using ArcGIS (ESRI, https://www.esri.com). We showed the distribution of *M. tuberculosis* genotype clusters if found within 1 km of one another to add epidemiologic plausibility. We excluded patients with missing residential geocoding from the spatial analysis.

### Sensitivity Analysis

To assess potential variation within genotype relatedness, we explored an alternative clustering definition to include 1 locus difference. For this sensitivity analysis, potential near matches (i.e., matched on all other loci results but with a nonmatched tandem number at the locus of interest) were considered genotype clusters. We excluded patients with isolates with missing or incomplete MIRU-VNTR results from the sensitivity analysis.

### Ethics Approval

This study was approved by the Institutional Review Boards of the US Centers for Disease Control and Prevention (#6291; Atlanta, GA, USA); Health Research and Development Committee, Botswana Ministry of Health and Wellness (Gaborone, Botswana); University of Pennsylvania (Philadelphia, PA, USA); and University of California, Irvine (Irvine, CA, USA). Participants provided written informed consent.

## Results

**Figure 2:**
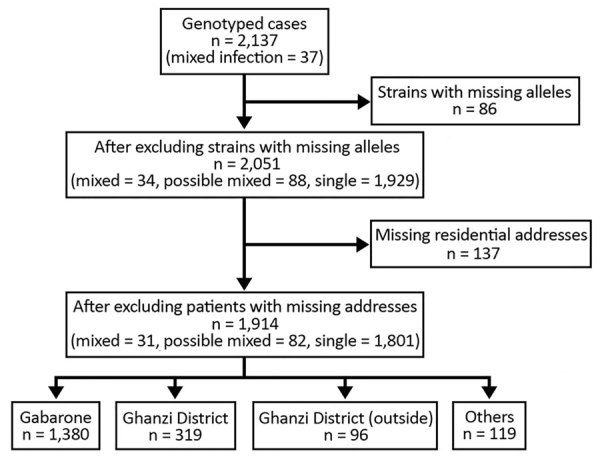
Flowchart of population-based, molecular epidemiology study (the Kopanyo Study) of mixed *Mycobacterium tuberculosis* strains, Botswana, 2012–2016.

A total of 2,137 patients were enrolled, of whom 1,130 (53%) were HIV positive (Table 1). After excluding patients with missing or incomplete MIRU-VNTR results (including 3 patients with mixed infection), we included 2,051 patients in the analyses (Figure 2). A total of 862 discrete genotyping MIRU-VNTR results were obtained (a more detailed strain analysis is available elsewhere [*15*]). We detected no evidence of laboratory cross-contamination events within sputum processing, culturing, DNA abstraction, or genotyping processing. All mixed infection patient isolates were processed on different days from isolates from other purported patients in the cluster.

**Table 1:** Characteristics of persons in a study to assess mixed-strain transmission of *Mycobacterium tuberculosis* (the Kopanyo Study), Botswana, 2012–2016

Characteristic	HIV status		
	Positive	Negative	Unknown
Total, no. (%), N = 2,137	1,130 (53)	948 (44)	59 (3)
Age, y, mean (± SD)	36.9 (10)	32.5 (16)	33.5 (14)
Sex, no. (%)			
M	558 (49)	584 (62)	43 (73)
F	572 (51)	364 (38)	16 (27)
Primary residential site, no. (%)			
Gaborone	828 (73)	565 (60)	43 (73)
Ghanzi District	109 (10)	215 (23)	8 (14)
Other Botswana, not in study region	116 (10)	108 (11)	0
Missing residential address	77 (7)	60 (6)	8 (14)
Previous tuberculosis history, no. (%)			
Yes	227 (20)	150 (16)	11 (19)
No	903 (80)	798 (84)	48 (81)
Infection status, no. (%)*			
Mixed	17 (2)	15 (2)	2 (4)
Possible mixed	50 (5)	35 (4)	3 (5)
Single	1,008 (93)	869 (94)	52 (91)
Different strains, no. (%)†	570 (66)	453 (53)	50 (6)

*After excluding 86 patients with mycobacterial interspersed repetitive unit–variable-number tandem-repeat results with missing alleles. †Total number of different mycobacterial interspersed repetitive unit–variable-number tandem-repeat results = 862.

Thirty-four (2%) patients had mixed infection, and 88 (4%) patients had possible mixed infection. Overall, we classified mixed infection in 23 (68%) patients as infection from a recently acquired strain and simultaneous reactivation of a remotely acquired strain, 7 (21%) as simultaneous reactivation of >2 remotely acquired strains, and 4 (12%) as >2 recently acquired strains (Table 2). Mixed infection in 27 (79%) patients involved recently acquired strains (Appendix Tables 1–3). The MIRU-VNTR results of 34 patients with mixed infection had a median of 7.5 loci (interquartile range 3–11) of multiple tandem repeats. The most prevalent MIRU-VNTR result in the population, MIRU identification no. [ID] 644 (n = 147 isolates), was not included in any genotype clusters with potential mixed infection transmission events. The second most prevalent strain, MIRU ID 382 (n = 81), matched with 2 genotype clusters involving patients with mixed infection (MIRU ID 838 and MIRU ID 970) (Appendix Table 1).

**Table 2:** Characteristics of 34 patients with mixed-strain *Mycobacterium tuberculosis* infection (the Kopanyo Study), Botswana, 2012–2016

Characteristic	No. (%)
Primary residential site	
Gaborone	22 (65)
Ghanzi District	7 (21)
Other Botswana	2 (6)
Missing residential address	3 (9)
HIV infection status	
Positive	18 (53)
Negative	14 (41)
Unknown	2 (6)
Transmission mechanism	
Recently acquired + recently acquired	4 (12)
Recently acquired + remotely acquired	23 (68)
Remotely acquired + remotely acquired	7 (21)
HIV infection & transmission mechanism	
Recently acquired + recently acquired	Positive: 1 (6); negative: 2 (14)
Recently acquired + remotely acquired	Positive: 12 (66); negative: 10 (72)
Remotely acquired + remotely acquired	Positive: 5 (28); negative: 2 (14)

**Figure 3:**
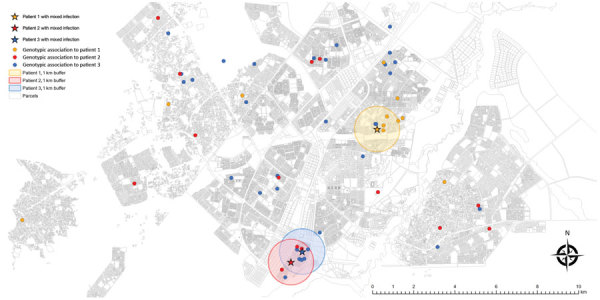
Potential spatial relationships (residence within 1 km of another patient) between patients with mixed-strain infection and with other genotype-clustered strains, Gaborone, Botswana, 2012–2016. Shown are location of patients with mixed *Mycobacterium tuberculosis* infection and other genotype-clustered cases in Gaborone. Each color represents each genotype cluster. The 1-km radius blue-shaded area from each mixed infection patient shows the neighborhood boundary. Three patients with mixed infection had potential spatial relationships with 3–6 other patients within the neighborhood.

**Figure 4:**
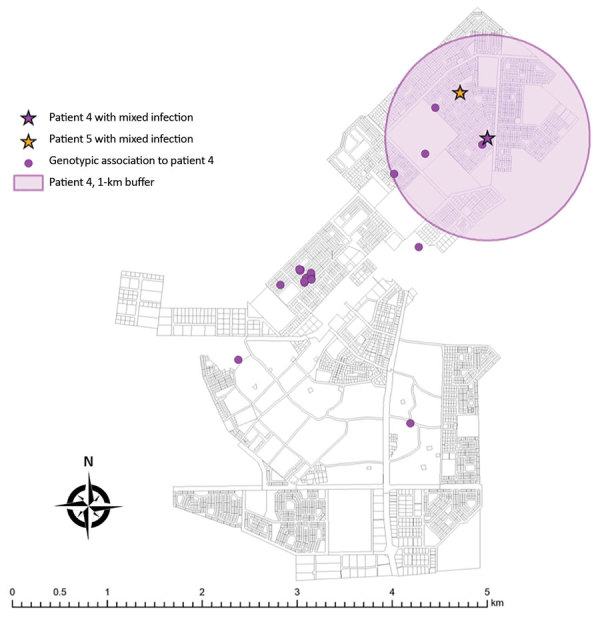
Potential spatial relationships (residence within 1 km of another patient) between mixed infection and other genotype-clustered cases, Ghanzi, Botswana, 2012–2016. Shown are locations of patients with mixed *Mycobacterium tuberculosis* infection and other genotype-clustered cases. Each color represents each genotype cluster. The 1-km radius blue-shaded area from each mixed infection patient shows the neighborhood boundary. Two patients with mixed infection were genotype-clustered and had a potential spatial relationship. (Their mycobacterial interspersed repetitive unit–variable-number tandem-repeat results were not exactly matched.)

After excluding additional 137 patients with no residential address (including 3 patients with mixed infection and 6 with possible mixed infection), we explored spatiotemporal transmission among 1,914 patients (Figure 2). We found 4 genotype clusters of mixed infection within 1 km of the location of patient with mixed infection as the center: 3 in Gaborone (Figure 3) and 1 in Ghanzi (Figure 4).

In sensitivity analysis, we allowed MIRU-VNTR patterns to differ by 1 locus, which changed the transmission category for 7 patients with mixed infection. Our main finding that the highest proportion (19 [51%]) of mixed infection occurred through a combination of genotype clustered and nonclustered strains did not change. The second highest proportion (10 [27%]) of mixed infection was a combination of multiple genotype clustered strains.

## Discussion

We describe genotype patterns consistent with hypothesized mixed infection transmission mechanisms, using a multiyear, population-based TB cohort. In our study, most patients with mixed infection (68%) had both recently and remotely acquired strains, suggesting recent transmission and simultaneous remote reactivation. Recent infection that progresses to disease might further compromise the immune system, leading to reactivation. A previous case study described a patient with mixed infection with an apparent triggering of a remote multidrug-resistant *M. tuberculosis* strain after recent exposure to a drug-sensitive strain (*16*). A similar phenomenon has been described for relapse of *Plasmodium vivax* malaria triggered by infection with *P. falciparum* (*17*).

Similarly, our findings suggest that most mixed infection transmission events included reactivation of remotely acquired strains triggered by recently acquired strains, implying that mixed infection may be affected by the force of infection in communities (*10*,*11*). We estimated the prevalence of each discrete MIRU-VNTR result as a proxy measure of force of infection in our study population. Contrary to our expectation, the 2 most prevalent strains (MIRU IDs 644 and 382) appeared in only 1 mixed infection transmission event. The dominate strain in the mixed infection was MIRU ID 838, which appeared 3 times. Further studies can show whether less transmissible strains outcompete other strains within the host to establish long-term persistence (*11*).

Our results add to the complexity of TB transmission dynamics in high TB prevalence settings (*7*). Current TB prevention strategies primarily focus on interrupting recent TB transmission through early detection and treatment of sputum smear–positive patients (*18*). Although interventions to interrupt transmission can reduce opportunities of exogenous re-infection and hence reduce the prevalence of mixed infection (*10*), our findings also imply the importance of treating latent TB infection to reduce the risk for mixed infection (*19*). No statistical association between HIV status—a proxy for reduced latency—and mixed infection (data not shown [odds ratio 1.15 (95% CI 0.79–1.68)]) also further supports the influence of remotely acquired strains in polyclonal transmission events. Our study alone might not be sufficient to generalize the results and emphasize reactivation. However, we envision further exploration of our suggested 3 transmission mechanisms in a setting where the transmission intensity is expected to be higher (e.g., high population density or dense slum area) and the role of reactivation is accounted for accordingly.

We added the spatial information to provide epidemiologic evidence of possible *M. tuberculosis* transmission. If patients whose isolates are in the same genotype cluster are spatially close to each other (i.e., within 1 km), they might be more likely to be in a transmission network than otherwise. This interpretation may be limited as we accounted only for the patients’ residential address as the spatial information, the close proximity set as 1 km was arbitrary, and the few TB clusters (and number of patients therein) may be missed if mixed infection is not included in transmission network reconstructions. However, our finding reconfirmed that TB transmission was ongoing in the community. A comprehensive molecular characterization of within-host *M. tuberculosis* diversity, as well as an attempt to temporally identify the primary source or index of transmission by comparing diagnosis times and the times of symptom onset (*20*), might be needed to fully capture TB transmission chains and accurately infer TB transmission (*21*,*22*).

Our results should be interpreted with caution. The prevalence of mixed infection was lower than in other studies (*9*,*23*) because of the method of molecular analyses. Although 24-loci MIRU-VNTR is a standardized molecular characterization tool and offers simple results that can be readily used to identify mixed infection (*7*,*24*), it has limited resolution to distinguish mixed infection from clonal heterogeneity or within-host bacterial microevolution (*9*,*25*,*26*). Different tools, such as whole-genome sequencing and 2 lineage-specific PCRs, might identify *M. tuberculosis* strains more sensitively and lead to a different dominating transmission mechanism if more patients with mixed infection were detected (*23*,*27*). In the meantime, we defined and analyzed possible mixed infection and mixed infection separately in an attempt to more conservatively differentiate mixed infection from within-host heterogeneity. Another limitation involves misclassification bias from detection sensitivity (*28*); that is, all potential genotyping matches depend on the sensitivity of the characterization method. The 24-loci MIRU-VNTR method is relatively sensitive and has high discriminatory power; however, it characterizes only part of the *M. tuberculosis* genome (*7*). Hence, we might have missed genetic heterogeneity present in loci not covered by this method (*12*). The prevalence of each *M. tuberculosis* strain also depended on the degree to which we captured all *M. tuberculosis* strains present in the community. Although our study was multiyear and covered a broad geographic area, some important patients in the transmission network could have been missed (e.g., their TB was diagnosed before the study period, they resided in areas not covered by the study, or they refused enrolment), leading to clustering misclassification. We recruited TB patients through both passive and active case finding (*13*) to increase coverage, but not every patient produced sputum, and not all sputum samples led to *M. tuberculosis* isolation or valid genotype results (*15*). This limitation might lead to missed transmission links (*18*,*21*). Given generally low bacillary load among children, the transmission mechanism would have been affected in a way that the role of reactivated strains was reduced if missing sputum samples had been successfully identified. On the other hand, by enabling multiple permutations of possible MIRU-VNTR results for mixed infection and possible mixed infection cases, MIRU-VNTR results with multiple alleles had more possible combinations and higher chance of matching with other genotypes. This finding may imply an imbalanced chance of being a member of a genotype cluster.

Future studies to investigate molecular profiles of *M. tuberculosis* with serial sputum collection, including nonrespiratory samples, and use of more sensitive and specific genome sequencing technologies, will be of interest to thoroughly assess possible transmission events leading to mixed infection. Despite the lower prevalence of mixed infection in the population in this study, the proposed mixed infection transmission mechanisms can be useful to characterize how similar or different mixed-infection transmission mechanisms would be across different settings with different burden of mixed infection.

Appendix Additional results for study of possible transmission mechanisms of sixed-strain *Mycobacterium tuberculosis*, Botswana, 2012–2016.
